# Supplementary material for: Limited application of reflective surfaces can mitigate urban heat pollution
Source: Nat Commun. 2021 Jun 9;12:3491. doi: 10.1038/s41467-021-23634-7 (PMC8190117; doi:10.1038/s41467-021-23634-7)
Supplement: Supplementary file 1 — Supplementary Information [file 41467_2021_23634_MOESM1_ESM.pdf]

Supplementary Information

Supplementary Figures

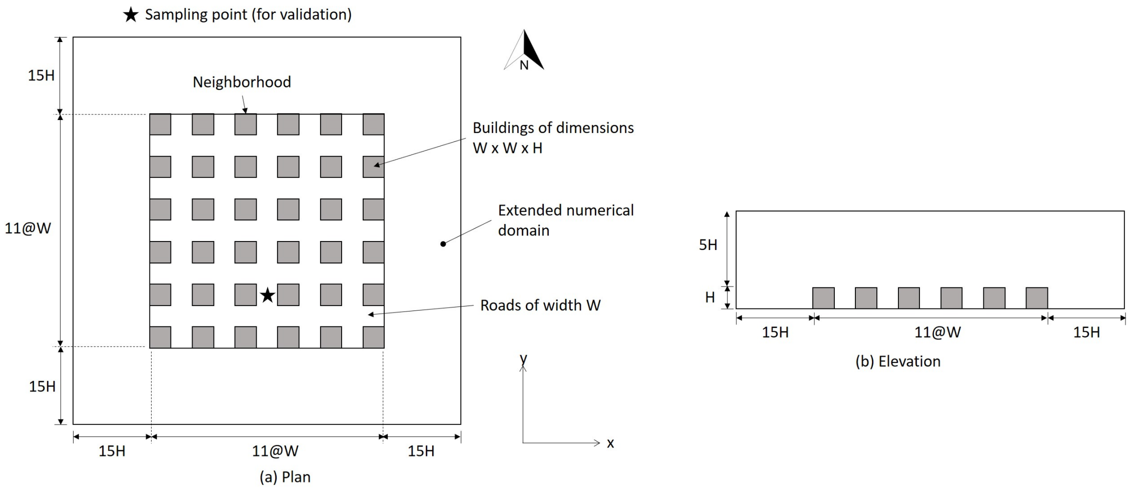

Supplementary Figure 1: Prototypical area for this study. a Plan view. b Elevation view.

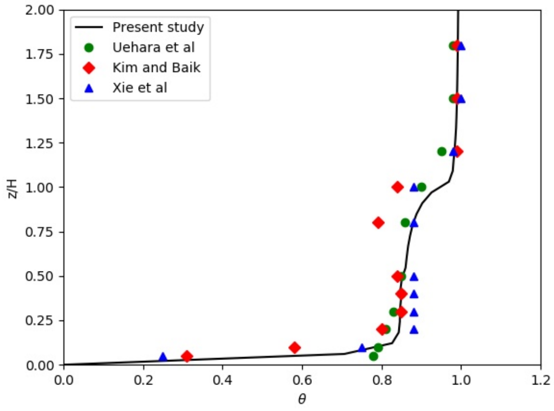

Supplementary Figure 2: Validation results.

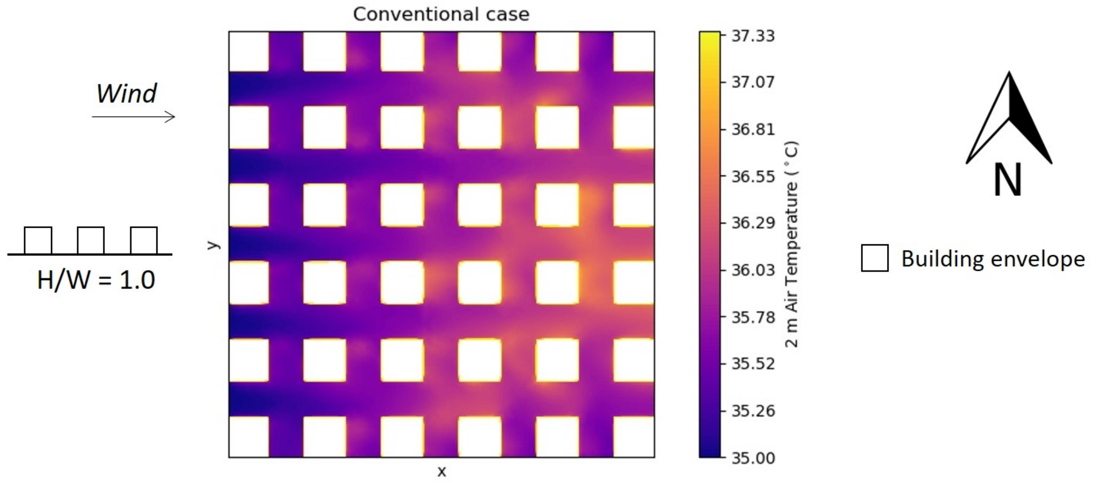

Supplementary Figure 3: 2 m air temperature for the Conventional case for westerly wind and H/W = 1.0.

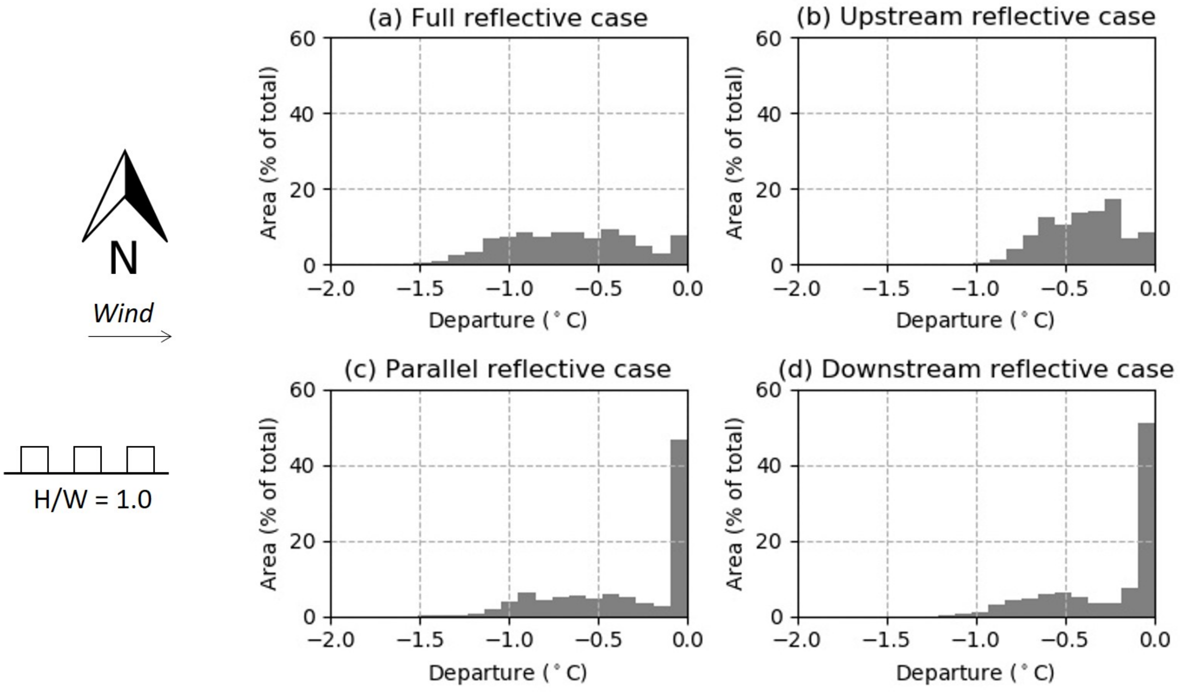

Supplementary Figure 4: Histogram of 2 m air temperature departure from the Conventional case relative to the total area for westerly wind and H/W = 1.0. a Full reflective case. b Upstream reflective case. c Parallel reflective case. d Downstream reflective case.

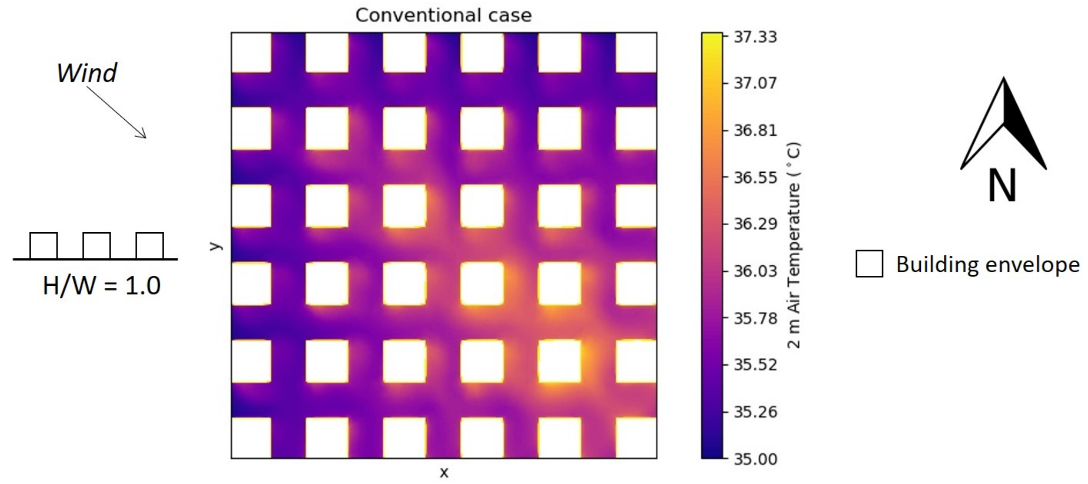

Supplementary Figure 5: 2 m air temperature for the Conventional case for northwesterly wind and H/W = 1.0.

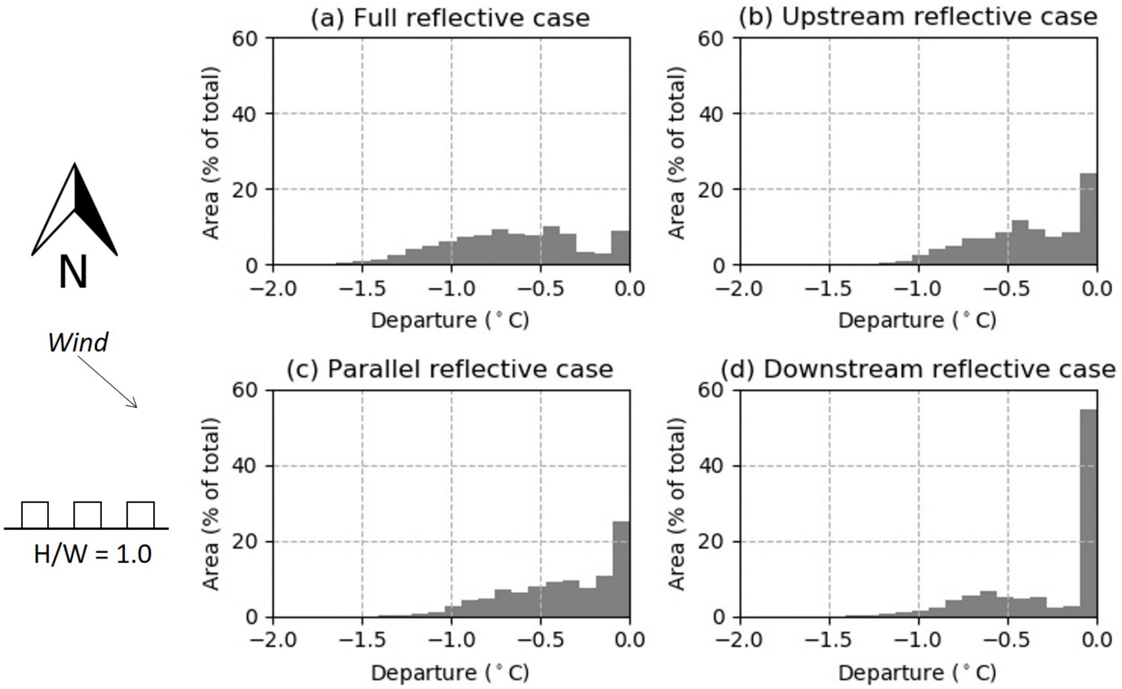

Supplementary Figure 6: Histogram of 2 m air temperature departure from the Conventional case relative to the total area for northwesterly wind and H/W = 1.0. a Full reflective case. b Upstream reflective case. c Parallel reflective case. d Downstream reflective case.

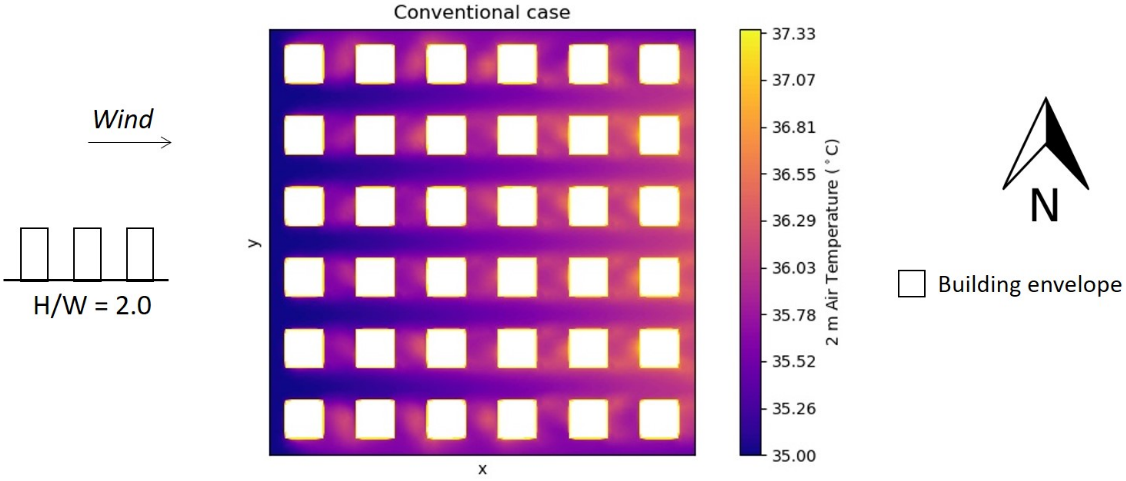

Supplementary Figure 7: 2 m air temperature for the Conventional case for westerly wind and H/W = 2.0.

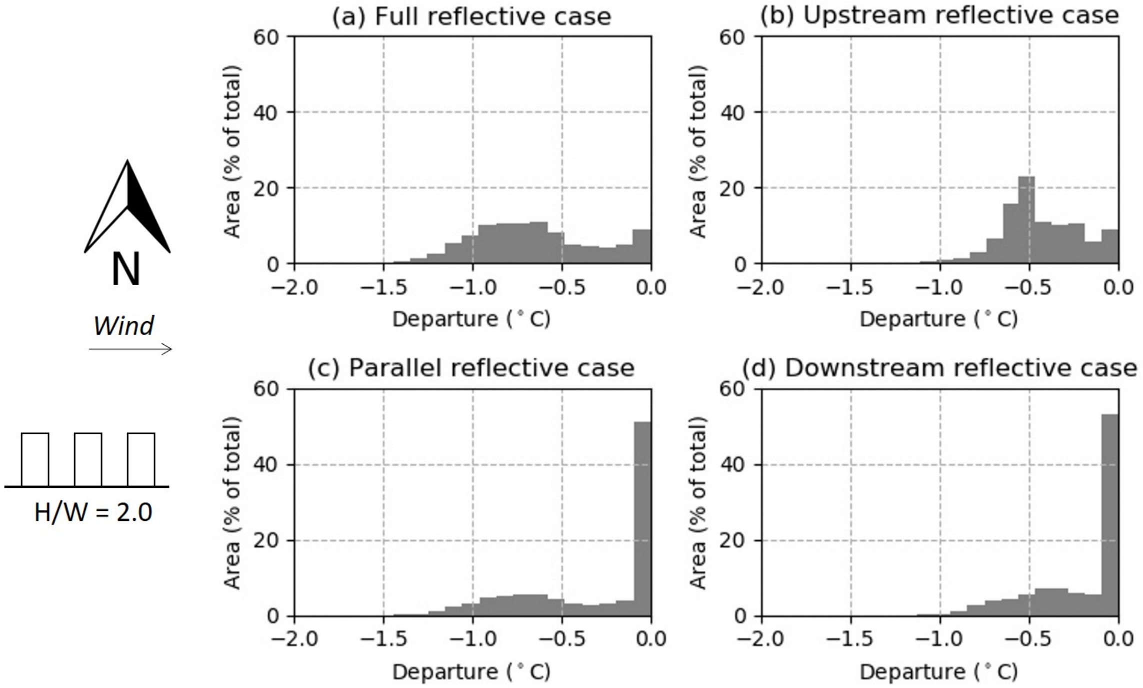

Supplementary Figure 8: Histogram of 2 m air temperature departure from the Conventional case relative to the total area for westerly wind and H/W = 2.0. a Full reflective case. b Upstream reflective case. c Parallel reflective case. d Downstream reflective case.

Supplementary Tables

Supplementary Table 1: Results of the convergence study

Mesh	Number of elements	C_d	Grid Convergence Index (%)
Coarse	157040	0.023	-
Medium	324280	0.024	16.0
Fine	840320	0.025	11.5

Supplementary Note 1: Numerical model

Consider a neighborhood of a city adjacent to either rural areas or other parts of the city. Wind blows into this neighborhood at a particular speed, direction, and temperature from these adjacent areas. Inside the neighborhood, the presence of roads and buildings leads to a non-uniform distribution of wind flow through advection. Furthermore, as sunlight heats the surfaces (roofs, walls, and roads) in the neighborhood, the air in turn is heated through convective heat transfer, establishing convective currents. The interaction between convective currents and wind flow advection within the neighborhood establishes a non-uniform air temperature field. The determination of heat pollution at the scale of a neighborhood requires the resolution of these variables within the neighborhood. CFD can resolve these variables by solving the governing equations of mass, momentum, and temperature fluxes of air within an urban domain for a given set of boundary conditions (wind speed, direction, and air temperature outside the neighborhood, as well as temperature of the surfaces).

The CFD model used in the present study resolves both the wind flow advection as well as convective heat transfer within the neighborhood. It does this by solving the 3D steady-state Reynold's Average Navier-Stokes (RANS) equations with Boussinesq approximation numerically. The RANS equations, shown in Supplementary Equations 1 and 2, respectively, denote conservation of mass and momentum, respectively, for incompressible flow. These account for wind flow advection and convection due to temperature differences. In addition, the RANS temperature Supplementary Equation is shown in Supplementary Equation 3, which resolves the 3D air temperature field. For a given set of boundary conditions of the incoming air and surfaces, these Supplementary Equations resolve the coupled wind speed and air temperature fields within a neighborhood, which can then be used to study heat pollution at a neighborhood scale.

$$\frac{\partial U_i}{\partial x_i} = 0 \tag{1}$$

$$U_j \frac{\partial U_i}{\partial x_j} = -\frac{\partial P}{\partial x_i} + (\nu + \nu_t)\frac{\partial}{\partial x_i}\frac{\partial U_i}{\partial x_i} - \beta(T - T_{ref})g_i \tag{2}$$

$$U_i \frac{\partial T}{\partial x_i} = (\alpha + \alpha_t)\frac{\partial}{\partial x_i}\frac{\partial T}{\partial x_i} \tag{3}$$

Here, U_i is the ith component of the RANS velocity in ms^{-1}, P is the kinematic pressure in $\mathrm{m}^2\mathrm{s}^{-2}$, ν is the kinematic viscosity of air that was assumed to be 1.6×10^{-5} $\mathrm{m}^2\mathrm{s}^{-1}$, ν_t is the turbulent viscosity calculated from a realizable k-ϵ closure model, $\beta = 3.3 \times 10^{-3}$ K^{-1} is the coefficient of thermal expansion of air, T is the RANS temperature, T_{ref} is a reference air temperature, g_i is the acceleration due to gravity (-9.81 ms^{-2}), $\alpha = Pr/\nu$ is the thermal diffusivity of air with Prandtl number $Pr = 0.70$, and $\alpha_t = Pr_t/\nu_t$ is the turbulent thermal diffusivity with turbulent Prandtl number $Pr_t = 0.85$.

Two salient features of the model may be noted from the Supplementary Equations. First, temperature and momentum were coupled through the buoyancy term at the end of Supplementary Equation 2, which is the so-called Boussinesq approximation. This is valid when expected temperature differences in the problem are within 4°C. This approximation is used to decrease computational time while still obtaining accurate solutions. Second, the RANS Supplementary Equations are steady-state and thus describe the system when it is at equilibrium with its boundary conditions. Practically, this represents an approximation in which the average wind speed and surface temperature at the system boundaries over a period of time (typically an hour) are set as boundary conditions, and the solution represents the average wind speed and air temperature over that time within the neighborhood. Both of these approximations have been widely used in urban CFD modeling, and were thus adopted in the present model as well.

The RANS Supplementary Equations and the temperature Supplementary Equation were solved numerically using a Finite Volume scheme implemented in the open-source solver OpenFOAM v5 [1]. Some other features of the model that decrease the computational time are discussed in the previous study [2].

Supplementary Note 2: Urban domain

The numerical model was used to resolve wind speed and air temperature fields in an urban neighborhood. For the present study, a prototypical urban neighborhood was developed, as shown in Supplementary Figure 1(a) and (b) in plan and elevation, respectively. It consisted of a 6×6 array of buildings of height H and square footprint $W \times W$, separated by roads of width W. The aspect ratio H/W could be varied to model neighborhoods of different densities, with a higher value representing a denser neighborhood [3].

In order to use the numerical model to solve the governing Supplementary Equations, the numerical domain had to be extended around the neighborhood in order to remove boundary effects from the solution of interest within the neighborhood. This extended domain was of length $15H$ laterally around the neighborhood and height $5H$ above the top of the buildings. These dimensions were based on best-practice guidelines for urban CFD modeling [4]. In this neighborhood, the roads, walls, and roofs had a higher temperature than the incoming air and therefore heated the air, while also modifying the wind flow. These were resolved using the model.

Supplementary Note 3: Convergence and validation

Before using the model to study the effect of reflective surfaces in the neighborhood, the model was first checked for convergence and validation. For both of these, data from wind tunnel experiments by Uehara et al. [5] was used. Those experiments were performed on a scaled urban neighborhood model similar to that shown in Supplementary Figure 1, with building height and inlet velocity selected so that Reynold's Number $Re = 3,500$, inlet temperature and surface temperature selected so that the Bulk Richardson Number $R_b = -0.21$, and aspect ratio $H/W = 1.0$. The same experimental conditions were replicated with the numerical model.

First, the domain was meshed using the extrusion technique developed by Van Hooff and Blocken [6] to yield a hex-dominant mesh. Three meshes with an increasing number of elements were developed, as summarized in Supplementary Table 1. For each mesh, the steady-state drag coefficient C_d, which is an integrated value representing the entire velocity field, was evaluated.

For all three meshes, C_d was quite similar. The Grid Convergence Index (GCI), which is a uniform way to report grid convergence considering mesh refinement proposed by Roache [7], is also reported. It was 16.0% for the Medium mesh and 11.5% for the Fine mesh, which are typical of urban CFD studies and generally considered adequate for convergence. To balance accuracy and speed, the Medium mesh was used for the rest of the study. For this mesh, the temperature profile at the sampling point, indicated with a star in Supplementary Figure 1, was extracted along the vertical coordinate z. The non-dimensional temperature profile θ is plotted in Supplementary Figure 2. On the same graph, experimental data from Uehara et al. [5] as well as data from two other numerical studies [8, 9] that also examined urban neighborhoods using CFD models under the same conditions, are plotted for comparison. There is good agreement between all these studies, thus validating the model.

Supplementary Note 4: 2 m air temperatures for Conventional case

For the lower-density building configuration (H/W = 1.0) with a westerly wind, the 2 m air temperature for the Conventional case is shown in Supplementary Figure 3. At the upstream end, the air temperature was not much higher than that of the incoming air as it was still being heated. However, the temperature increases further downstream, and was highest in the wake of the buildings. The air temperature rose to about 37.5°C, representing heat pollution to the extent of 2.5°C. Previous studies have shown that for every 1°C increase in air temperature, residential water consumption for a typical family home can increase by about 2,000 liters per month [10], monthly electricity consumption in the hottest months can increase by up to 8.5% [11], and the potential for thermal discomfort and heat-related morbidity can also increase significantly [12]. Thus, heat pollution to the extent of 2.5°C can be quite significant.

The lower-density building configuration (H/W = 1.0) was simulated with a northwesterly wind to examine the effect of wind direction. The 2 m air temperature for the Conventional case is shown in Supplementary Figure 5. As compared to the lower-density with westerly

wind case discussed above, the air temperatures were similar but were skewed along the new wind direction, with the air being gradually heated to about 37.5°C as it flowed through the neighborhood, once again representing 2.5°C of heat pollution in the wake of downstream buildings.

A higher-density building configuration (H/W = 2.0) was simulated with a westerly wind to examine the effect of urban density. The 2 m air temperature for the Conventional case is shown in Supplementary Figure 7. Similar to the previous two configurations, the air was steadily heated as it flowed through the neighborhood, with downstream areas experiencing up to 2.5°C higher temperatures. However, the distribution of the temperature was different, with the higher temperatures more concentrated in downstream areas and in the wake of the buildings. This is caused by a higher rate of advection as compared to diffusion on account of the more constricted canyon geometry. A relative decrease in diffusion as compared to advection leads to less mixing and hence a less uniform air temperature distribution.

Supplementary Note 5: 2 m air temperature departures

For each of the cases, the departures were also visualized in the form of histograms. The histograms for the lower-density building configuration (H/W = 1.0) with a westerly wind are shown in Supplementary Figure 4. For the Full reflective case (Supplementary Figure 4(a)), the histogram was relatively flat and broad, indicating that the 2 m air temperature decreased over a large part of the neighborhood, with some parts experiencing a greater decrease than others. In comparison, for the Upstream reflective case (Supplementary Figure 4(b)), the histogram was relatively taller and narrower, indicating that there was a smaller decrease in 2 m air temperature over a smaller area of the neighborhood. Finally, both the Parallel and Downstream reflective cases (Supplementary Figure 4(c) and (d) respectively) were similar, with a sharp peak around zero and a relatively narrow curve after that. This shows that for both cases, a large part of the area experienced very little cooling.

For the lower-density building configuration (H/W = 1.0) with a northwesterly wind, the histograms are shown in Supplementary Figure 6. The Full reflective case (Supplementary Figure 6(a)), the histogram was again relatively flat and broad, indicating widespread cooling albeit at a high cost. Unlike the previous configuration, however, the Upstream and Parallel reflective cases (Supplementary Figure 6(b) and (c) respectively) were similar, with a slight peak around zero and relatively narrow after that, indicating that a larger area of the neighborhood experienced no cooling as compared to the Full reflective case, although there was still fairly widespread cooling. On the other hand, the Downstream reflective case (Supplementary Figure 6(d)) had a sharp peak around zero and a relatively narrow curve beyond, indicating a lack of any cooling effect for a large part of the neighborhood.

Finally, for the higher-density building configuration (H/W = 2.0) with a westerly wind, the histograms are shown in Supplementary Figure 8. Like the two previous configurations, the Full reflective case (Supplementary Figure 8(a)) had a relatively flat and broad shape, indicating widespread cooling. The Upstream reflective case (Supplementary Figure 8(b)), however was relatively narrow, with a slight peak around -0.5°C, indicating that the cooling effect, while still present, was more spatially concentrated. Finally, the Parallel and Downstream reflective cases (Supplementary Figure 8(c) and (d) respectively) had a sharp peak

around zero with a relatively narrow curve beyond, which shows that a large part of the neighborhood experienced no cooling effect.

Supplementary References

[1] Henry G Weller, Gavin Tabor, Hrvoje Jasak, and Christer Fureby. A tensorial approach to computational continuum mechanics using object-oriented techniques. *Computers in physics*, 12(6):620–631, 1998.

[2] Sushobhan Sen and Jeffery Roesler. Wind direction and cool surface strategies on microscale urban heat island. *Urban Climate*, 31:100548, 2020.

[3] Juan A Acero, Jon Arrizabalaga, Sebastian Kupski, and Lutz Katzschner. Urban heat island in a coastal urban area in northern spain. *Theoretical and applied climatology*, 113(1-2):137–154, 2013.

[4] Jorg Franke, Antti Hellsten, K Heinke Schlunzen, and Bertrand Carissimo. The cost 732 best practice guideline for cfd simulation of flows in the urban environment: a summary. *International Journal of Environment and Pollution*, 44(1-4):419–427, 2011.

[5] Kiyoshi Uehara, Shuzo Murakami, Susumu Oikawa, and Shinji Wakamatsu. Wind tunnel experiments on how thermal stratification affects flow in and above urban street canyons. *Atmospheric Environment*, 34(10):1553–1562, 2000.

[6] T Van Hooff and Bert Blocken. Coupled urban wind flow and indoor natural ventilation modelling on a high-resolution grid: A case study for the amsterdam arena stadium. *Environmental Modelling & Software*, 25(1):51–65, 2010.

[7] Patrick J Roache. Perspective: a method for uniform reporting of grid refinement studies. 1994.

[8] Jae-Jin Kim and Jong-Jin Baik. Urban street-canyon flows with bottom heating. *Atmospheric Environment*, 35(20):3395–3404, 2001.

[9] Xiaomin Xie, Chun-Ho Liu, and Dennis YC Leung. Impact of building facades and ground heating on wind flow and pollutant transport in street canyons. *Atmospheric Environment*, 41(39):9030–9049, 2007.

[10] Subhrajit Guhathakurta and Patricia Gober. The impact of the phoenix urban heat island on residential water use. *Journal of the American Planning Association*, 73(3): 317–329, 2007.

[11] Matheos Santamouris, Constantinos Cartalis, Afroditi Synnefa, and Dania Kolokotsa. On the impact of urban heat island and global warming on the power demand and electricity consumption of buildingsa review. *Energy and Buildings*, 98:119–124, 2015.

[12] R Sari Kovats and Shakoor Hajat. Heat stress and public health: a critical review. *Annu. Rev. Public Health*, 29:41–55, 2008.
